# Analysis of the optical performance of intraocular lenses using profilometric measurements

**DOI:** 10.1007/s00417-024-06628-1

**Published:** 2024-09-17

**Authors:** Juan J. Miret, Vicente J. Camps, Celia García, Maria T. Caballero, Juan M. Gonzalez-Leal

**Affiliations:** 1 Group of Optics and Visual Perception. Department of Optics, Pharmacology and Anatomy, University of Alicante, San Vicente del Raspeig, Alicante, Spain; 2 Department of Condensed Matter Physics. Faculty of Sciences, University of Cadiz, Cadiz, Spain

**Keywords:** Optical quality, Multifocal Intraocular lenses, Extended Depth of Focus, Pupil size, Modulation Transfer Function, Decentration, Tilt

## Abstract

**Purpose:**

The aim of this study was to develop a methodology, based on profilometer measurements to assess the optical behaviour of Intraocular Lenses (IOls). The “Modulation Transfer Function through-object” (MTF through-object) based on vergence object displacement was calculated for different pupil sizes and pseudophakic eyes. Tilt and decentration were also analysed in a realistic cornea eye model.

**Methods:**

For comparison between the different IOLs, an optical quality criterion based on a minimum value the MTF through-object and the recognition of simulated vision optotypes was introduced. Five IOLs were used in this study: Tecnis Eyhance, Mini Well, Tecnis Symfony, Tecnis Synergy and RayOne EMV.

**Results:**

The technique was validated with previous methodologies. A general narrowing of the through-object MTF curve compared to the through-focus MTF curve was shown, resulting in greater distances between near and intermediate points and less depth of field around the far peak. The comparison between the IOLs showed that variations in corneal aberrations, pupil size and decentration caused relevant changes in IOL performance. A decrease of the SA produced a hypermetropic shift of the far focus between + 0.3 D and + 0.4 D. Most of IOLs worsen the optical quality as pupil size increased, even the MTF through-object shape changed. Decentration was an important factor in IOL implantation, causing a significant change in MTF through-object shape in most of IOLs.

**Conclusions:**

This study highlights the need to evaluate pre-operative patients for corneal aberrations and pupillary size to have the best optical success after cataract surgery in multifocal or extended depth of focus IOLs.

**Key messages:**

***What is known***

- MTF(Modulation Transfer Function) through-focus curves (calculated in image space by moving the detector plane) can be obtained from optical bench assembly or from commercial devices.
- Recently, some studies proposed to characterize the lens surface design based on the profilometric measurements

***What is new***

- A novel methodology based on profilometer measurements to assess the optical behaviour of Intraocular Lenses (IOls) was shown.
- The “Modulation Transfer Function through-object” based on vergence object displacement was introduced in order to analyse five premium IOLs.
- MTF through-object curve is more appropriate for studying clinical behaviour, as it provides further near and intermediate points distances and lower depth of focus around far peak compare to MTF through-focus curves.
- The optical behaviour of the five IOLs can vary considerably depending on the eye model and pupil size.
- The effect of tilt and decentration on the MTF through-object the IOLs was analysed.

**Supplementary Information:**

The online version contains supplementary material available at 10.1007/s00417-024-06628-1.

## Introduction

Lens surface manufacturing control has become a key factor in ensuring the best optical and clinical characteristics of multifocal intraocular lenses (MIOLs). Parameters as Strehl Ratio, Modulation Transfer Function (MTF), MTF through-focus curves, Point Spread Function (PSF), Area under MTF (MTFa), etc., are used to evaluate the optical quality [1–6]. These parameters can be obtained from optical bench measurements and/or simulations with optical system design software [1, 5, 7–10]. Usually, the MTF through-focus curves can be obtained from optical bench assembly or from commercial devices. The basic principles of these measurements are set out in ISO standards and the corresponding points of the curves are obtained in image space by moving the detector plane[11–13]. However, if the detector is in a fixed position the MTF through-focus curves could be measured by changing vergence in object space, either by placing lenses in front of the eye model or by means of Badal lenses through-focus [13]. In fact, there are some studies that have induced vergence changes in object space to calculate MTF through-focus curves[14–16], such as those using visual simulators or Badam systems. This variation of the MTF through-focus calculation, based on object displacement, would be conveniently called "MTF across the object", as the calculations are referenced to the object space.

Recently, some studies proposed to characterize the lens surface design based on the profilometric measurements, [17–20]. Specifically, a methodology based on the surface topography of the IOLs (both, refractive as diffractive) allowed to estimate if the surface profile was spherical, aspherical, or incorporated higher orders terms [17].

The aim of this work was to characterise the optical behaviour of IOLs based on surface profilometric measurements as well as their possible clinical implications. The optical parameter used for this purpose was the MTF through-object curve. Following the ISO standards to compare between the IOLs, the study of the IOL performance considering 3 mm and 4.5 mm exit pupil sizes and two eye models was conducted. These two eye models were based on aberrated and non-aberrated corneas, with only the primary spherical aberration considered in the aberrated model [11, 12]. However, in order to approach to a more real clinical perspective, since real corneas may have more higher order aberrations, a third corneal model with up to 4th order aberrations was included in the study. Consistent with this idea, the effect of tilt and decentration was also studied.

To our knowledge, no study was conducted with the combination of profilometer measurement and the calculation and analysis of MTF through-object curve. Therefore, in a first step, the proposed methodology was compared with previous ones (as based on optical bench or commercial devices) to establish the reliability of the methodology.

## Material and methods

### Intraocular lenses

Five IOLs were used in this study, two enhanced monofocal IOLs (Tecnis Eyhance and RayOne EMV), a bifocal IOL (Tecnis Symfony) and two extended depth of focus IOLs, one based on refractive optics (Mini Well) and one based on diffractive optics (Tecnis Synergy).

The Mini Well IOL (SIFI, Lavinaio, Italy) uses a high order aspheric surface design [9] divided into three different annular zones whose middle zones have SA with opposite signs [1, 21]. Tecnis Eyhance, (Johnson & Johnson Vision, Inc), has an spherical posterior surface that provided a negative SA of − 0.27 μm and anterior high order aspherical surface design [9]. The Tecnis Symfony IOL, (Johnson & Johnson Vision, Inc) combines diffractive and refractive technologies. This IOL has an aspheric anterior surface that provides a negative SA of − 0.27 μm and a fully diffractive and achromatic echelette posterior surface [9, 22]. In the Tecnis Synergy IOL (Johnson & Johnson Vision, Inc), the biconvex optic has a wavefront-designed aspheric anterior surface that also provides a negative SA of − 0.27 μm and a diffractive posterior optic[9, 23, 24]. The RayOne EMV (Rayner Intraocular Lenses Limited, Worthing, UK) is an aspheric monofocal IOL with an increased positive SA but with neutral aberration in the periphery [25].

### Surface measurement method

The measurement method and surface smoothing calculation process were detailed in the previous report [17]. The surface topography of the IOLs was measured by using a multimode optical profilometer (Zeta Instruments, model Z 300). The three-dimensional (3D) image of the surface along the diameter of the lens was obtained by confocal grid structured illumination. The smoothed profile was performed using routines and algorithms written in Matlab (MATLAB, The MathWorks, Natick, MA). We used an application programming interface (API) to link Zemax (ZEMAX OpticStudio, ZEMAX LLC.) with Matlab, and then we performed the entire simulation process, from raytracing to subsequent analysis of the results.

### Pseudophakic physiological eye model simulation and MTF through-object curve calculation process

An ocular physiology eye model based on Liou-Brenan eye model was used this study [26], because it will provide more relevant responses for objects at finite distances and better approximation to clinical interpretation as Norrby et al. stated [13]. The cornea proposed by this eye model was configured to contribute only the primary spherical aberration (${c}_{0}^{4}$= + 0.256 µ m), leaving the rest of the aberrations at 0 µ m [26]. This ${c}_{0}^{4}$ value approached to primary spherical aberration average cornea of the eye [27]. These cornea parameters were used to simulate the Eye Model 1consisting of the cornea and the IOL under study. However, ISO standards also propose an aberration-free cornea model [11, 12]. With this corneal model it is possible to compare the effect of the aberrations introduced by different IOLs, since the cornea does not contribute any aberrations. This condition was obtained from Liou-Brenan cornea eye model by changing the asphericity of the first corneal surface (see Table 1). With this aberration-free cornea, Eye Model 2 was constructed. However, as commented above, a more realistic cornea should be proposed in order to analyse the effect of other high order aberrations (see Table 2). Based on the study by Atchinson et al. [27], corneal averages of higher order aberrations up to 4th order were incorporated into the eye model (see Table 2). This model was called Eye Model 3. The effects of decentration and tilt on the imaging quality of IOLs have been widely studied and several studies have reported the postoperative tilt and decentring values of the IOLs[25, 28–30]. Eppig et al.[31], established average values for tilt and decentration by comparing several studies. In general, the mean decentration in the studies was 0.30 mm ± 0.16 (SD) (range 0.00 to 1.09 mm) and the mean tilt, 2.62 ± 1.14 degrees (range 0.20 to 8.17 degrees). Based on these results, the effect of a 0.5 mm decentration and 5º tilt on the MTF of the through object was analysed. This study was performed for the more realistic cornea (Model 3) and for a standard photopic pupil size of 3 mm.

**Table 1:** Corneal parameters of Liou-Brenan eye model [26]

	Radius (mm)	Asphericity (Model 1)	Asphericity (Model 2)	*n*_*c*_ *(*546 nm)	Corneal Thickness	(${c}_0^4$) Total Primary Spherical Aberration (Model 1)	(${c}_0^4$) Total Primary Spherical Aberration (Model 2)	Other High order aberrations (Model 1 and 2)
Anterior cornea	7.77	–0.18	–0.57	1.376	0.50 mm	+0.256 μm	0 μm	0 μm
Posterior cornea	6.40	–0.60	–0.60	1.336

**Table 2:** Corneal high order aberrations used in the Model 3 [26, 27]

	${C}_{3}^{-3}$ (µm)	${C}_{3}^{-1}$ (µm)	${C}_{3}^{+1}$ (µm)	${C}_{3}^{3}$ (µm)	${C}_{4}^{-4}$ (µm)	${C}_{4}^{-2}$ (µm)	${C}_{4}^{0}$ (µm)	${C}_{4}^{+2}$ (µm)	${C}_{4}^{+4}$ (µm)
Model 3	-0.034	+ 0.083	-0.073	+ 0.034	+ 0.007	0	+ 0.256	-0.011	-0.029

The MTF through-object curve was obtained by calculating the MTF value for 50 cycles/mm, in image space and changing the object vergence. As known, the MTF value for 50 cycles/mm is comparable to the evaluation of Visual Acuity with optotypes of 20/40 in white light (30 cpd) [32]. The defocuses used for through-object curve ranged between -3.5 D and + 1.5 D, so that the defocus of 0 D corresponded to far vision.

Calculations were conducted using standard settings featuring a monochromatic light (546 nm) and two pupil sizes of 3 mm and 4.5 mm placed in IOL plane[11, 12]. The axial length was estimated for 3 mm pupil size and finding the retina position where the value of the MTF was maximum for an object in the infinite.

## Results

### Comparison of MTF through-focus curves from profilometer measurements

**Fig. 1:**
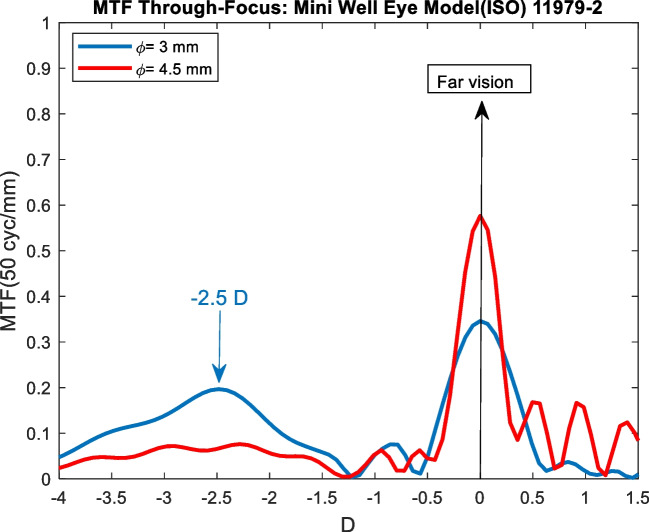
MTF through-focus curves of the Mini Well IOLs implementing the profilometer measurements in the eye model proposed in the (ISO) 11,979–2 standard [12] and for a pupil sizes of 3 mm (blue line) and 4.5 mm (red line) in IOL plane

Most of techniques used to analyze optical quality of LIOs are based on optical bench by implementing the ISO standards[11, 12]. These studies provide the MTF through-focus curves by moving the detector through-focus. MTF through-focus curves obtained from profilometer measurements and from optical bench were compared using the profilometric measurements of a known IOL (Mini Well,) in a ISO eye model [12]. Dominguez et al. using a specific device (PMTF, Lambda-X, Belgium) obtained the MTF through-focus curves of these IOL using an aberration-free eye model of the instrument in accordance with the Standardization (ISO) 11,979–2 [33]. The MTF through-focus curves of the Mini Well from profilometric measurements for 3 mm and 4.5 pupil sizes are shown in Fig. 1. The shape of the MTF through-focus curves were almost identical to that obtained by Domínguez et al. In addition, similar results were showed by Belluci et al.[34] but using a different eye model configuration. Same comparisons were performed for the rest of the IOLs studied in this paper and the same results were concluded independent the eye model used [22, 25, 35–37].

### Differences between the MTF through-object and MTF through-focus

**Fig. 2:**
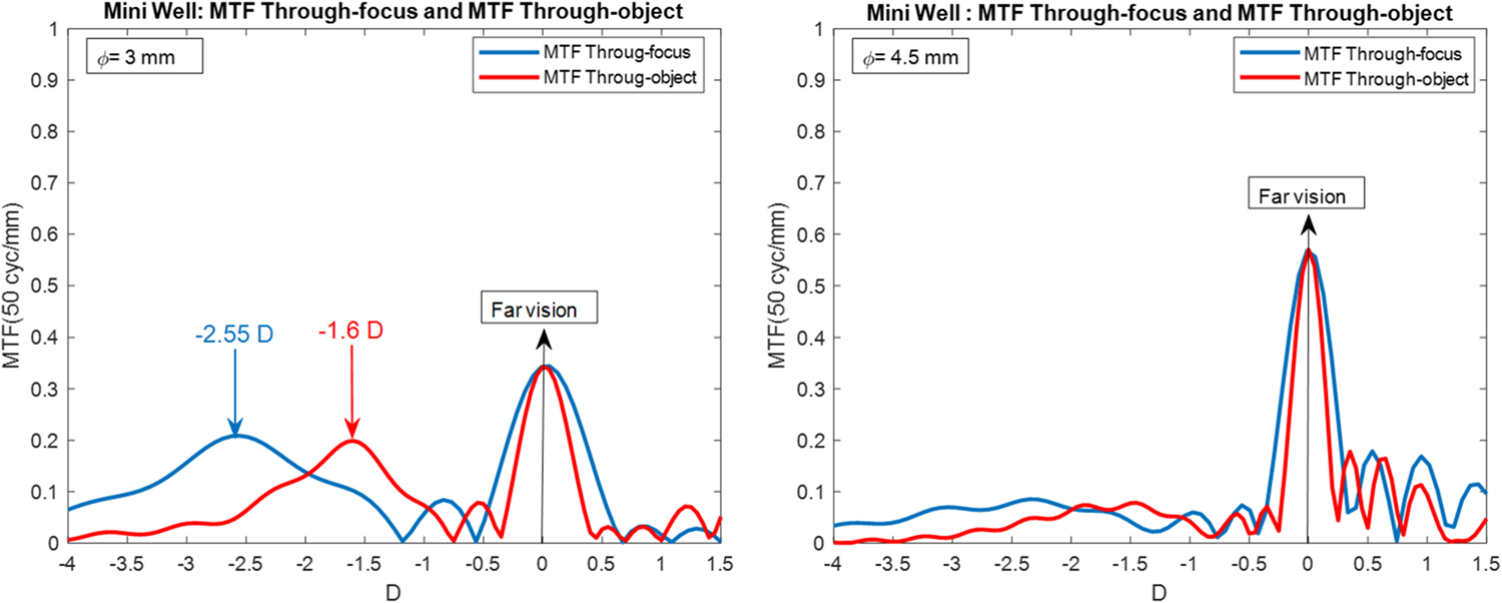
Comparison of the MTF through-focus (blue line) and MTF through-object (red line) obtained for the Mini Well and for 3 mm pupil size (left) and 4.5 mm pupil size (right)

As explained above, the MTF through-object curve was obtained by changing the vergence object instead the detector plane. The reference plane of MTF through-object is the corneal vertex while in MTF through-focus calculation is the exit pupil of the IOL. In any case, both curves will be related using power translation equations from one plane to the other. Figure 2 shown the MTF through-object and MTF through-focus curves for the Mini Well calculated using the model 2 (aberration-free cornea).

As seen in Fig. 2, significant differences were found. Although the shape of MTF through-focus and through-object were similar, the MTF through-object was narrowed compared to the MTF through-focus. For Mini Well and 3 mm pupil size, MTF through-focus curve provided a peak at -2.55 D and the MTF through-object at -1.6 D (0.95 D of difference). For 4.5 mm pupil size both peaks were located at far vision. An important consequence of this result is the fact that de zone of depth of focus was reduced when MTF through-object was considered. This conclusion was also observed around the far peak. These differences could be important when translating them into distances. The same conclusions were reached if other eye models were considered.

### MTF through-object and its interpretation

**Fig. 3:**
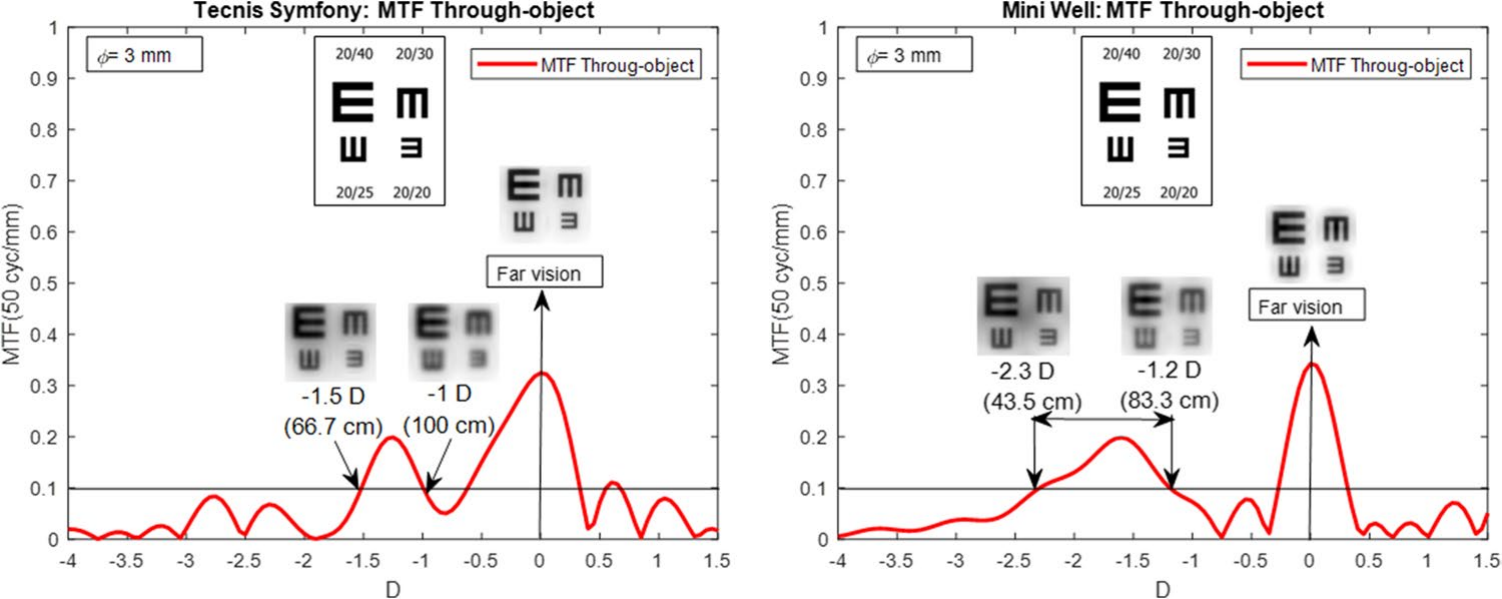
Interpretation of the MTF through-object for the Tecnis Symfony (left) and the Mini Well (right) for 3 mm pupil size. Far, intermediate and near points when MTF (50 cycles/mm) value is 0.1 are shown with the corresponding simulated vision optotypes

Since the MTF through-object is representing the object space, the dioptric distances (and therefore the distances) are measured from corneal vertex without the use of any transformation. In order to be able to compare the possible quality of vision between different IOLs from MTF through-object curves, a qualitative criterion based on a minimum value of MTF for 50 cycles/mm of 0.1 was proposed. This value was established after several simulations with different IOL and finding that it provided a simulated optotype image with a recognizable visual acuity of at least 20/25 (see Fig. 3). This criterion does not mean that equal or better visual acuity can be obtained with lower MTF values, but, this limit will be valid to compare intermediate and near vision zones for different IOLs providing a good approximation of how different IOLs perform in these zones.

As example, MTF through-object curves obtained from profilometric measurements for 3 mm pupil size of the Tecnis Symfony and the Mini Well IOLs are shown in Fig. 3. As seen, both IOLs provided good far vision as the optotypes were clearly recognizable. In addition, following the described criterion, a near point in 43.5 cm (-2.3 D) would be observed for Mini Well IOL and in 67.7 cm (-1.5 D) for the Tecnis Symfony, and an intermediate point in 83.3 cm (-1.2 D) for Mini Well and 100 cm (-1 D) for the Tecnis symphony.

With these criteria, the comparison between different IOLs can be better understood and conclusions such as that the Mini Well will provide a greater depth of focus and consequently a closer near point than the Tecnis Symfony can be affirmed.

### MTF through-object curves depending on the eye model and pupil size

**Fig. 4:**
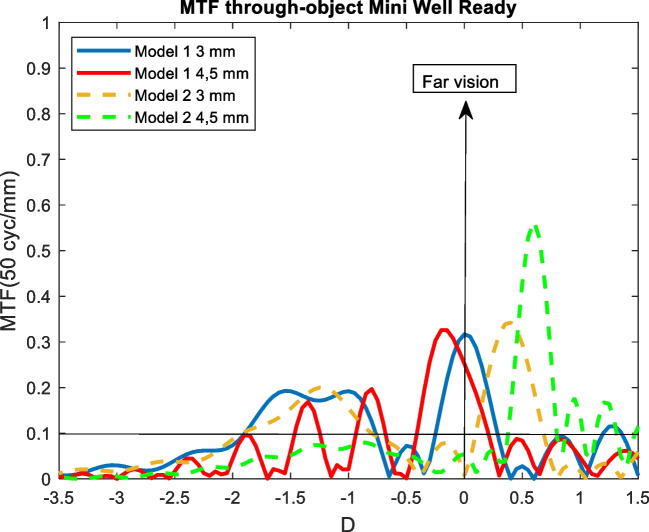
MTF through-object curves of the Mini Well IOL for two eye models and two pupil sizes

**Fig. 5:**
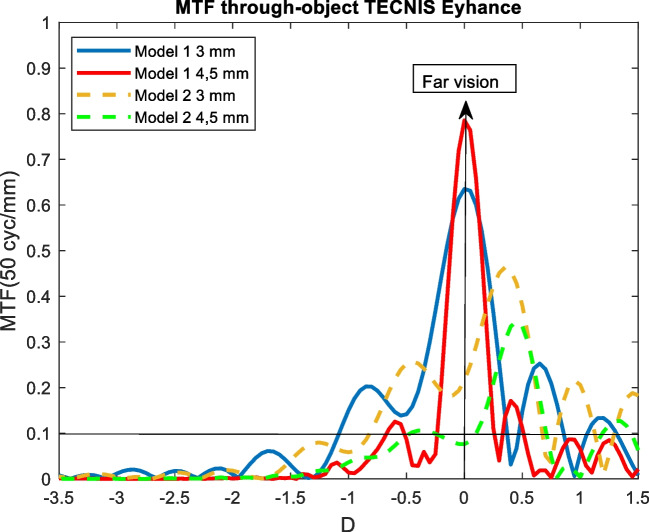
MTF through-object of the Tecnis Eyhance IOL for two eye models and two pupil sizes

**Fig. 6:**
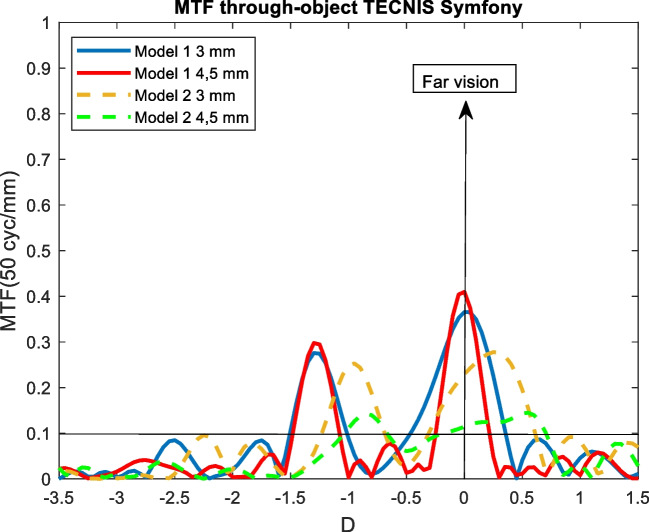
MTF through-object of the Tecnis Symfony IOL for two eye models and two pupil sizes

**Fig. 7:**
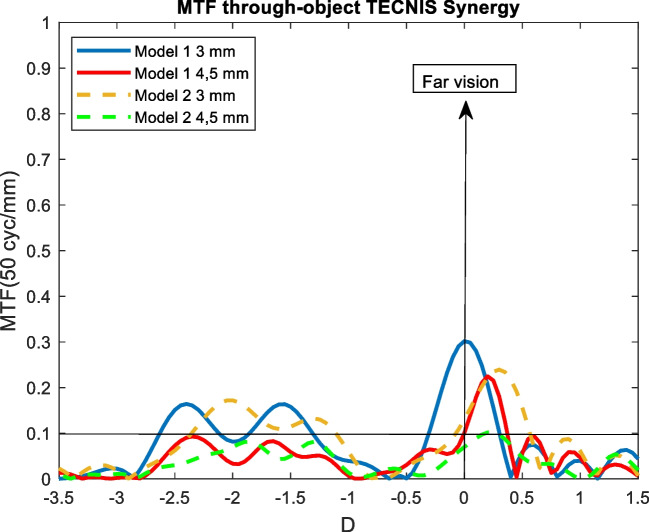
MTF through-object of the Tecnis Synergy IOL for two eye models and two pupil sizes

**Fig. 8:**
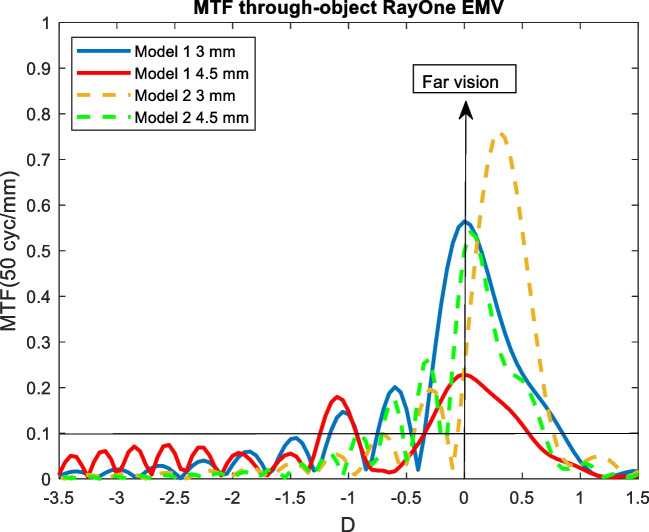
MTF through-object of the RayOne EMV IOL for two eye models and two pupil sizes

Figures 4, 5, 6, 7, and 8 showed the MTF though-Object curves for the five IOLs. As explained in methodology section, model 1 corresponded to an aberrated cornea of + 0.258 μm and it was represented with continuous lines, model 2 corresponded to an aberration-free cornea eye model and it was represented with dashed lines. Blue line corresponded to 3 mm pupil size and red line to 4.5 mm pupil size. In addition, a line indicated the minimum value of 0.1 for the MTF.

### Mini Well

#### Model 1

As seen in Fig. 4, aberrated cornea eye model for 3 mm pupil size provided a good far vision and a dioptric distance of 2 D up to near point. An extended DOF between -2 D (50 cm) and -0.75 D (1.33 m) was obtained. For 4.5 mm pupil size a -0.15 D myopic shift of the far focus was observed and the extended DOF converted to three different peaks at -1.85 D (54 cm), -1.35 D (74 cm) and -0.8 D (1.25 cm).

#### Model 2

When an aberration-free cornea eye model and 3 mm pupil size were considered, the MTF through-object curve was very similar to the model 1. The far focus shown a hypermetropic shift of + 0.4 D. The near point was at -1.95 D, therefore the dioptric distance between far and near vision increased to 2.35 D (instead 2 D in model 1). For 4.5 mm pupil size the Mini Well behaved as monofocal IOL with far focus centred in + 0.6 D (see Fig. 4).

### Tecnis Eyhance

#### Model 1

For model 1 and 3 mm pupil size, the Tecnis Eyhance IOL showed a far focus and some extend DOF around it up to -1.1 D (90.9 cm). When 4.5 mm pupil size was considered, the IOL behaved as a monofocal IOL (see Fig. 5).

#### Model 2

When aberration-free cornea eye model and 3 mm pupil size were considered, a general worsening effect and a far focus hypermetropic shift of + 0.35 D were observed. The dioptric distance between the far focus and the near point was slightly increased up to 1.2 D. For 4.5 mm pupil size the IOL only showed a peak at + 0.45 D with a decrease in optical quality (see Fig. 5).

### Tecnis symfony

#### Model 1

As seen in Fig. 6, for both pupil sizes the Tecnis Symfony behaved as a bifocal IOL, with an intermediate point at -1.25 D (80 cm).

#### Model 2

There was a hypermetropic shift of + 0.3 D for both pupil size with a general worsening in optical quality. For 3 mm pupil size, the dioptric distance between the two peaks was the same than for model 1 (1.2 D). For a pupil size of 4.5 mm, the optical quality deteriorated considerably (see Fig. 6).

### Tecnis Synergy

#### Model 1

Tecnis synergy showed an extended DOF from -2.6 D (38.5 cm) to -1.3 D (76.9 cm), however, a worsening of the optical quality (MTF lower than 0.1) around 2 D (50 cm) was observed. For 4.5 mm pupil size, there was an overall worsening of the optical quality and only a peak centred at + 0.25 D was observed (see Fig. 7).

#### Model 2

For 3 mm pupil size, a hypermetropic shift of the curve of + 0.3 D was obtained. For 4.5 mm pupil size the MTF through-object decreased below 0.1 (see Fig. 7).

### RayOne EMV

#### Model 1

For 3 mm pupil size, RayOne EMV IOL showed an extended DOF around the far focus up to 1.5 D. When 4.5 mm pupil size was considered, decrease of the optical quality was observed (see.

Figure 8).

#### Model 2

At 3 mm pupil size the far focus is shifted + 0.3 D and a DOF of 0.8 D around the far focus was observed compared to model 1. At 4.5 mm pupil size, the optical quality decreased (see Fig. 8) and hyperopically shifted.

### MTF through-object curves depending on the tilt and decentering

The effect of tilting and decentering in IOLs was studied for the Model 3 and 3 mm pupil size. Most IOL designs are thought to performance optimally when the cornea has positive spherical aberration and a photopic pupil size of 3 mm. Specifically, the effect of a 0.5 decentration in the y-direction and a five degree (5°) of tilt was analyzed. In following figures, the MTF through-object of the model 3 (blue line) is compared with the model 3 (orange line), with a decentering of 5 mm (green line) and a tilt of 5º (pink line).

### Mini well

**Fig. 9:**
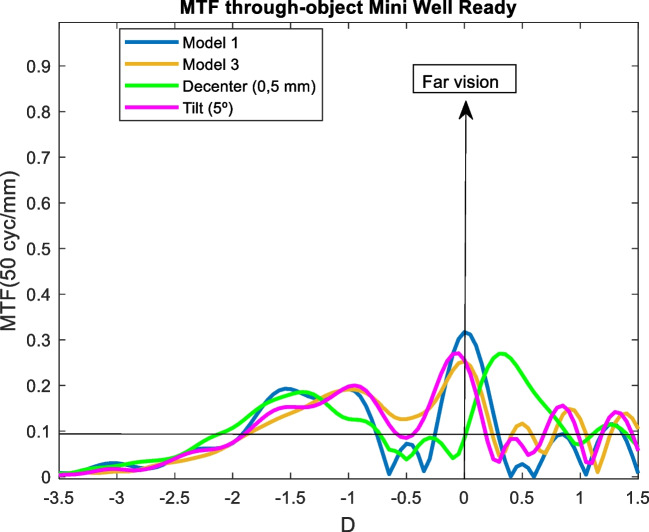
MTF through-object curves for a 3 mm pupil size of the Mini Well IOL. Model 1 (blue line) with a decentration of 5 mm (green line) and a tilt of 5º (pink line), and Model 3 (orange line)

As can be seen in Fig. 9, decentration was the factor that modified the MTF through-object curve the most. Increasing the aberrations (Model 3) caused a general worsening effect but maintained the shape. Finally, the tilt was well tolerated by the IOL as the curve remained practically the same. Similar results (but with MTF through-focus) regarding the tilt were obtained by Belluci et al. [34].

### Tecnis eyhance

Clearly, decentration was the factor that most affected IOL optical performance. Far vision was the most impaired although the curve remained above 0.1 for far and intermediate vision. Both Model 3 and the tilt showed very similar curves.

### Tecnis symfony

Decentration was the factor that most influenced IOL behaviour. While the bifocality of the IOL was observed for Model 3 and tilt, decentration caused an EDOF effect but with a deterioration of the optical quality.

### Tecnis synergy

Model 1, Model 3 and tilt showed the same results. Decentration caused a deterioration for distance vision, but for the EDOF zone all the curves remained similar. Our results were comparable tho those obtained by Can et al., except for the tilt, where we found a higher worsening [38].

### RayOne EMV

The MTF through-object is hardly affected by decentration and tilt when a realistic eye model is used.

## Discussion

A methodology based on profilometer measurements was demonstrated to be useful to assess the optical quality of IOLs. This methodology was comparable with optical bench-based methodologies as shown in Fig. 1. In addition, a proposed qualitative criterion based on simulated vision optotypes was introduced in order to compare the results of different IOLs. This qualitative criterion consisted in obtaining a recognizable optotype at least of 20/25 for a MTF through-object value of 0.1. This limit allowed to determine near, intermediate and far points with acceptable quality of vision and compare them between different IOLs. As seen in Fig. 2 the MTF through-object curve (based on object vergence displacement) shown a general narrowing compared with the MTF through-focus curve. This narrowing provided that the near and intermediate points distances were further (up to 1 D) and the DOF around the far peak was lower. The differences between the two curves will depend on the eye model, pupil size and IOL type, but curve narrowing always will occur. In this context, the MTF through-object curves will provide more realistic results since visual acuity is measured, for example, in object space.

Another result of this study was that the optical behaviour of IOLs depended on the eye model and pupil size. In the aberrated cornea eye model (model 1), the Mini Well showed an extended DOF from 50 cm to 1,33 m. As seen in Fig. 4, the decrease in spherical corneal aberration (from + 0.258 μm in model 1 to 0 μm in model 2) for 3 mm pupil size showed a hypermetropic shift of + 0.4 D of all the curve. Ruiz-Alcocer et al. found similar results, as they indicate a shift 0.5[32]. However, if the hypermetropic shift was compensated, this could be an advantage, since the dioptric distance between far and near vision would increase to 2.35 D and the near point would be at 44 cm. When 4.5 mm pupil size was considered, in model 1 the IOL showed oscillations around the DOF zone and in model 2 behaved as a monofocal IOL. As seen previously, the shape of the curves of the model 2 was very similar to obtained by Dominguez et al., but the dioptric distance between far and near foci was higher (3,45 D instead 2,5 D in our results) [33]. The differences were due to the differences between MTF through-focus and through-object curves.

The Tecnis Eyhance behaved like an enhanced monofocal IOL for 3 mm pupil size independently the eye model (see Fig. 5). However, a 0.35 D shift between both models was found. When 4.5 mm pupil size was considered the enhancement trend decreased and it became a monofocal IOL. In model 2 the optical quality was poorer. The results with model 1 were very similar to those obtained by Schmid et al. with the MTF throughfocus[25], but showing a lower curve widening that indicated a nearer intermediate point and less DOF around far focus. In addition, our study showed new results when the free aberration cornea was considered since the optical quality decreased and a hypermetropic shift up to 0.6 D for a pupil size of 4.5 mm was observed.

Tecnis Symfony behaved as bifocal IOL independently on the pupil size with an intermediate point at 80 cm (see Fig. 6). Model 2 showed poorer optical quality for the pupil size of 4.5 mm. The results for model 2 were comparable to obtained by Dominguez [33] and Chae [22] and, for model 1 with the obtained by Son [37], Chae [22] and Calatayud [39]. However, in both studies, the dioptric distance between the 2 peaks was approximately 1.75 D (57.1 cm for the intermediate point), whereas we obtained 1.25 D (80 cm) with the MTF through-object.

Tecnis Synergy for 3 mm pupil size and model 1 provided a near point at 38.5 cm and an intermediate point at 76.9 cm (see Fig. 7). Similar result was obtained in aberration-free cornea and 3 mm pupil size but with an hypermetropic shift of + 0.3 D. However, as pupil size increased, the optical quality decreased significantly. Only a far peak was found in both models and, MTF values were always equal or lower than 0.1. Recently, Labuz et al. [36] reported MTF though-focus considering a monochromatic green light with an aberration-free cornea and a polychromatic light with an aberrated cornea. Our MTF through-object curves for 3 mm pupil size and both models were very similar but we obtained farther near and intermediate points. For 4.5 mm, the results with model 1 were close and different with model 2 because we obtained an inferior optical quality. In addition, they didn’t report the hypermetropic shift.

RayOne EMV behaved like an enhanced monofocal IOL except for 3 mm pupil size and model 2 (see Fig. 8). RayOne EMV IOL showed an extended DOF around the far focus up to 1.5 D in model 1 and up to 0.8 D in model 2. These results were in agreement those obtained by Schmid et al. [25] who reported the MTF through-focus curves following the ISO-2 eye model. As previous results, we obtained lower DOF around the far peak and consequently farther intermediate points.

It appears that the best performance of all IOLs is obtained with a 3 mm pupil size and a standard average aberrated cornea (+ 0.256 μm). When the corneal spherical aberration decrease to 0 μm (or becomes less positive), a hyperopic shift is generally observed.

Patients with a cornea with spherical aberration near to standard and pupil size of approximately 3 mm, will accept all IOLs for distance vision. The best quality of vision is provided by the Tecnis Eyhance and the RayOne IOLs (called enhanced monofocal IOLs), as the MTF through-object is higher. However, these IOLs provide further intermediate points (higher than 1 m compared to the rest of IOLs). If the near vision is a requirement for the patient, the Mini Well and the Tecnis Synergy IOLs are the best options. These IOLs also provide good intermediate point of vision like the Tecnis Symfony, which shows better quality for this point. In patients with larger pupil sizes, the IOLs changed the optical performance. The Tecnis Synergy was the most affected because distance vision could be compromised.

In patients with less positive spherical aberration than the average cornea [27], most of the IOLs maintained their properties after accounting for the hyperopic shift. However, if the patient has a larger pupil size, the optical quality of the IOL will be very low and certainly not accepted by the patient.

**Fig. 10:**
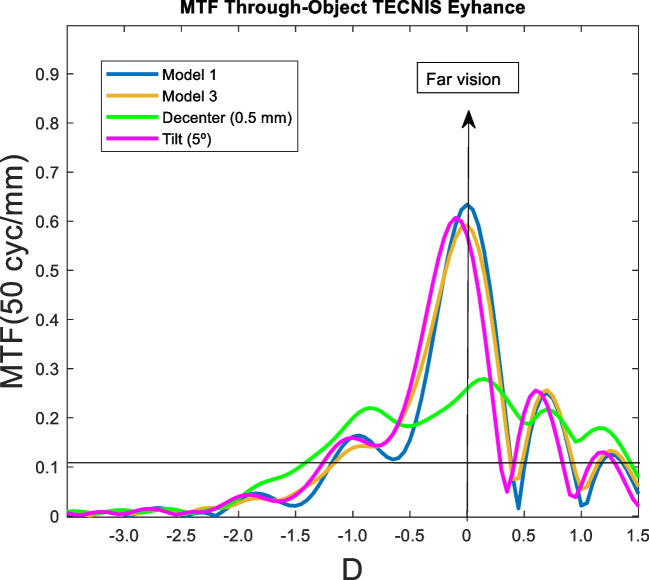
MTF through-object curves for a 3 mm pupil size of the Tecnis Eyhance IOL. Model 1 (blue line) with a decentration of 5 mm (green line) and a tilt of 5º (pink line), and Model 3 (orange line)

**Fig. 11:**
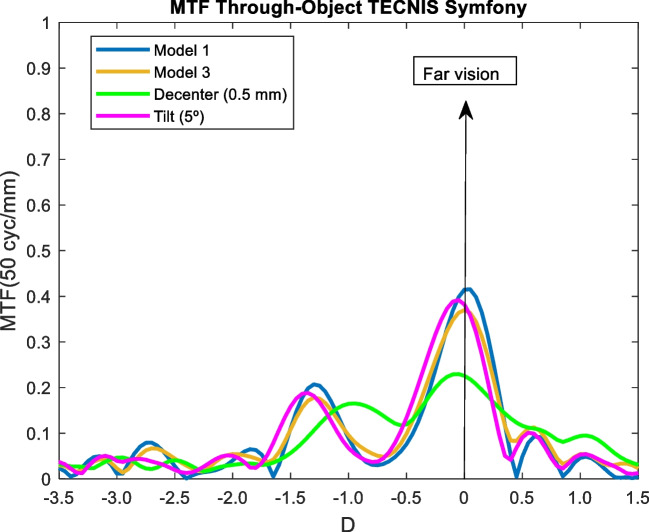
MTF through-object curves for a 3 mm pupil size of the Tecnis Symfony IOL. Model 1 (blue line) with a decentration of 5 mm (green line) and a tilt of 5º (pink line), and Model 3 (orange line)

**Fig. 12:**
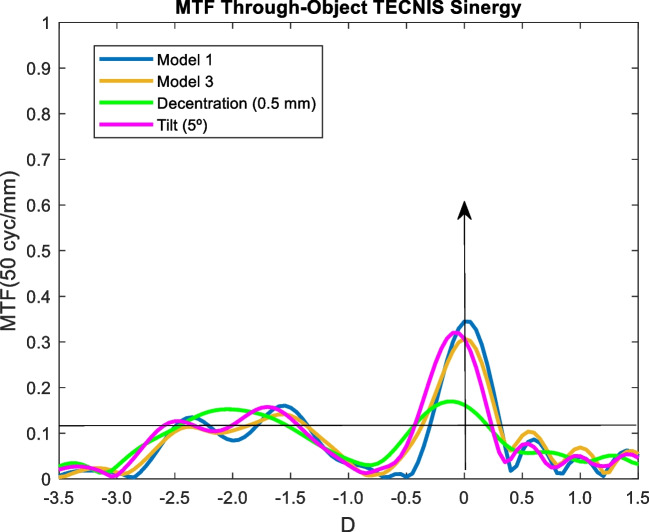
MTF through-object curves for a 3 mm pupil size of the Tecnis Synergy IOL. Model 1 (blue line) with a decentration of 5 mm (green line) and a tilt of 5º (pink line), and Model 3 (orange line)

**Fig. 13:**
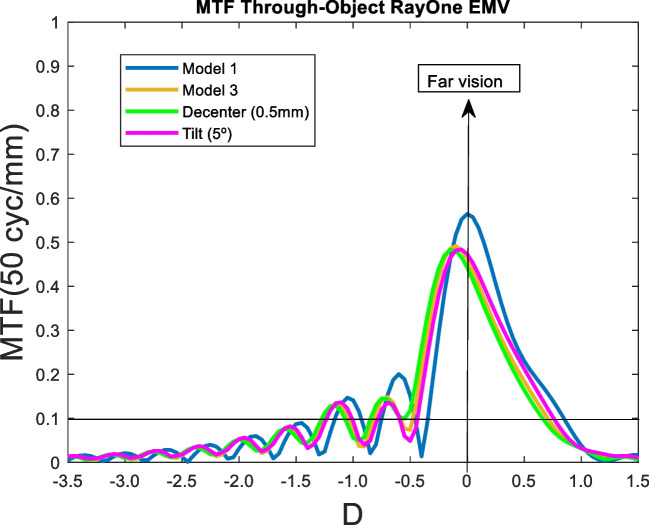
MTF through-object curves for a 3 mm pupil size of the RayOne EMV IOL. Model 1 (blue line) with a decentration of 5 mm (green line) and a tilt of 5º (pink line), and Model 3 (orange line)

The effect of increasing high order aberrations was analysed by the Model 3. As seen in Figs 9, 10, 11, 12 and 13. the effect was negligible for all IOLs. For tilt, the results were very similar because the MTF through-object was hardly modified. Decentration was the factor that most affected the optical behaviour of the IOLs. A significant decrease of the MTF through-object was observed at far vision, except for the Mini Well IOL. In addition, for the Tecnis Eyhance and RayOne EMV IOLs an EDOF effect was observed as the MTF through-focus curves were wider. Similar behaviour was observed with the Tecnis Symfony, whose bifocality was changed and became less defined. Both the Mini Well and Tecnis Synergy IOLs maintained the same shape despite the decentration, but with lower optical quality. This deterioration was less pronounced for the Mini Well IOL. To our knowledge, this is the first time that the effect of tilt and decentration on the MTF through-object has been studied in these IOLs.

## Conclusions

A methodology based on profilometer measurements proved to be comparable with other methodologies for assessing the optical quality of IOLs. By means of this methodology, the MTF through-object, based on the vergency object change, was proposed to analyze the optical behavior of IOLs. We demonstrated a general narrowing in the shape of the curves compared to the MTF through-focus and, consequently, farther near and intermediate points and a smaller DOF around the far peak.

The results showed that variations in corneal aberrations, pupil size and decentration caused relevant changes in IOL behavior. All IOLs showed the best optical performance for Model 1 and 3 mm pupil size. This result is consistent with the fact that the IOLs are designed to take into account the average value of the primary spherical aberration of the cornea [27]. Moreover, the addition up to 4th order aberrations (model 3) did not significantly affect the optical behaviour, indicating that the primary spherical aberration of the cornea was the aberration that needs to be considered more. This assumption was supported by the fact that a decrease in SA (in our study from + 0.258 μm to 0 μm) generally produced a hypermetropic shift of the far focus between + 0.3 D and + 0.4 D. Most of IOLs worsen the optical quality as pupil size increased due to the increase in aberrations, even the MTF through-object shape changed. Therefore, it is important for the ophthalmologist to know the specifications of IOLs in terms of the aberrations they produce and the pupil for which they are designed, as both corneal aberrations and the patient's average pupil size can alter the optical behaviour of the implanted IOL. Specifically in this study, patients with an average cornea and pupil size of approximately 3 mm, will be good candidates for all IOLs if only good far vision is required, but, being enhanced monofocal IOLs which provide best optical quality because the MTF through-object curve was higher compared to the rest of IOLs. However, the Mini Well and Tecnis Synergy IOLs are the best options for near and intermediate performance. Tecnis Symfony could be a good option for intermediate vision but not for near vision. If the patient has larger pupil size the performance of the IOLs changed worsening the optical quality at the intermediate and near points locations. Although in patients with less positive spherical aberration, most of the IOLs maintained their properties (considering a hyperopic shift), the increase in pupil size becomes critical as the optical quality of the IOL is more compromised.

Finally, decentration has been shown to be an important factor in IOL implantation, resulting in a significant change in the MTF through-object shape with the exception of the Mini Well IOL and RayOne EMV.

All these results could be of great importance when multifocal IOLs are to be implanted in patients with previous refractive surgery. As known, the study of the effect of ocular aberrations in surgery has become increasingly important. Several studies have attempted to address the behaviour of IOLs under different aberrated eye conditions, specifically corneal aberrations as eyes with [5, 36] and without any previous refractive surgery [25, 36, 40]. As our study showed, the same optical solution (i.e. the same IOL) may behave differently depending on the patient's corneal aberrations or pupillary size. In conclusion, the results highlighted the importance of controlling corneal aberrations and pupillary size in patients undergoing multifocal or extended depth of focus IOLs implantation. MTF through-object curves could be more convenient to assess the clinical performance of these IOLs since most of clinical parameters, visual acuity at near or intermediate points, are measured in object space.

## Supplementary Information

Below is the link to the electronic supplementary material.

Supplementary file1 (PDF 337 KB)

## Data Availability

The datasets generated and/or analysed during the current study are not publicly available due to the IOLs are protected by patent, but are available from the corresponding author on reasonable request.
