# Supplementary material for: Analysis of the optical performance of intraocular lenses using profilometric measurements
Source: Graefes Arch Clin Exp Ophthalmol. 2024 Sep 17;263(2):451–65. doi: 10.1007/s00417-024-06628-1 (PMC11868146; doi:10.1007/s00417-024-06628-1)
Supplement: Supplementary file 1 — Supplementary file1 (PDF 337 KB) [file 417_2024_6628_MOESM1_ESM.pdf]

MTF Through-focus curve

The MTF is measured with the IOL placed in a model eye. The model eyes proposed by the standard consists of a wet-cell which is filled with saline solution and considers two artificial cornea approaches, free-aberrated or aberrated. The aberrated eye version introduces a cornea with spherical aberration, to better mimic the human eye. The model eye is mounted on the optical bench for generating the through-frequency MTF curves at different apertures for the images formed by the far power and by each near power. ISO standards indicates that the measures shall be made for different powers with the lens on-axis, decentered and tilted and focusing to give maximum modulation ratio for 50 *cyc/mm* in each case. Furthermore, a graph of the through-focus MTF response performance at 50 *cyc/mm* of the MIOL in the model eye and for different apertures shall be shown. The standard indicates that first step is to obtain the focus position that gives the maximum MTF at 50 *cyc/mm* for an object at infinity, and then measure the MTF at positions in image space corresponding to increasingly closer object distances up to that at 20 cm[1]. These measurements can be performed by displacing the microscope along the axial axis (see figure 8)

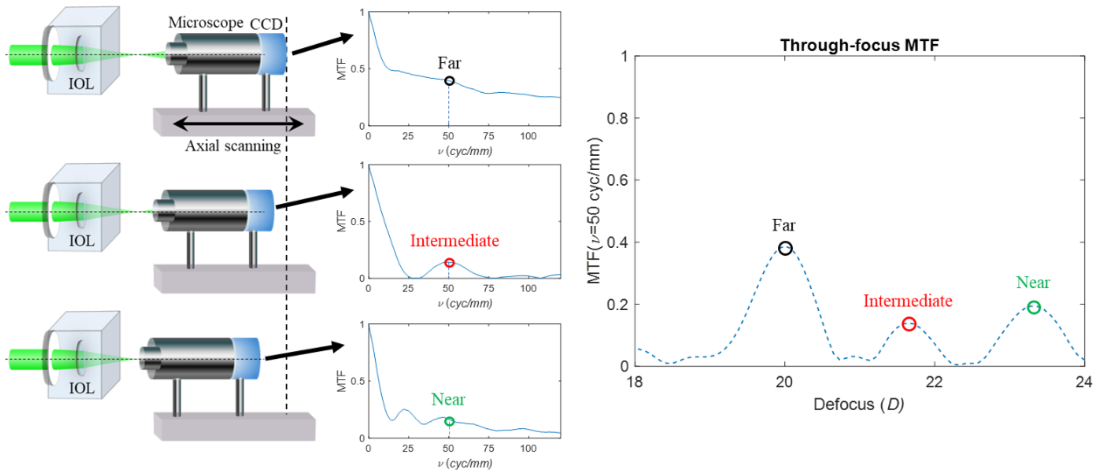

Figure 8: Schematic illustration depicting the process of deriving the MTF through-focus curve from the through-frequency MTF calculation along the axis for a specific frequency (50 *cyc/mm*).

The through-frequency MTF is obtained for the far focus and a its value for a specific frequency (for example 50 *cyc/mm*). This condition corresponds to the black point depicted in the graphs. Afterwards, the through-frequency MTF is obtained for different planes (moving the microscope) and the specific frequency value is always selected for each plane. That is the way the through-focus MTF curve is calculated. figure 8 shows three peaks representing to the far focus (black point), intermediate focus (red point) and near focus (green point) of a trifocal IOL.

Through-focus MTF at $50\ cyc/mm$ is commonly employed to assess and contrast the effectiveness of MIOLs.
